# Coach–Athlete Attachment and the Subjective Well-Being of Athletes: A Multiple-Mediation Model Analysis

**DOI:** 10.3390/ijerph17134675

**Published:** 2020-06-29

**Authors:** Jiaxi Peng, Jiaxi Zhang, Luming Zhao, Peng Fang, Yongcong Shao

**Affiliations:** 1 School of Psychology, Beijing Sport University, Beijing 100084, China; pengjx880124@hotmail.com; 2 Department of Political Theory, Xi’an Research Institute of High Technology, Xi’an 710086, China; zhangjiaxifmmu@126.com; 3 HSBC Business School, Peking University, Shenzhen 518055, China; lumingzhao@pku.edu.cn; 4 Department of Military Medical Psychology, Air Force Medical University, Xi’an 710032, China

**Keywords:** coach–athlete attachment, subjective well-being, perceived coach support, self-esteem

## Abstract

The current study aims to explore how coach–athlete attachment affects the subjective well-being (SWB) of athletes and is primarily focused on the confirmation of the mediating roles of athletes’ perceived coach support and self-esteem in the relationship between them. A total of 179 Chinese athletes participated in this study, in which they responded to questions comprising a coach–athlete attachment scale, a perceived coach support measurement, the Rosenberg self-esteem scale, and SWB measures. The results suggest that both attachment anxiety and attachment avoidance significantly predict SWB in athletes. The effects of attachment anxiety on SWB are partially mediated by perceived coach support and self-esteem, and the effects of attachment avoidance on SWB are completely mediated by perceived coach support and self-esteem. Moreover, a chain mediating effect was found: coach–athlete attachment → perceived coach support → self-esteem → SWB. These findings extend the conclusions of prior reports and shed light on how coach–athlete attachment influences the athlete’s well-being.

## 1. Introduction

Subjective well-being (SWB) is a subjective evaluation of happiness, and consists of life satisfaction, as well as positive and negative affect experiences [1,2]. SWB can be used to assess the general quality of life and reflect the psychological health condition and developmental level of individuals [3]. Among the different influencing factors of SWB, attachment has been increasingly studied [4,5], and refers to emotional associations with intimate people [6,7]. According to the theory of attachment, security is a fundamental human need and is closely related to healthy and fully-developed people [7]. Over the years, many studies have provided evidence that supports the relationship between attachment and SWB [8,9,10]. Overall, existing research suggests that available and supportive attachment is associated with positive affect and satisfaction with life. However, there has been little research that has explored the relationship between attachment and SWB for athletes.

Brennan et al. divided adult attachment into two dimensions: attachment avoidance and attachment anxiety [11]. The former refers to the feeling of inadaptation caused by intimacy with an attached subject, as well as the degrees of psychological and emotional independence, and the latter is defined as the degree of worry about being separated from or abandoned by the attached subject [11,12]. The two dimensions can be categorized into high and low levels; the attachment style, including low attachment anxiety and low attachment avoidance, is called secure attachment, while all other combinations are considered insecure attachment [13,14]. As has been previously reported, attachment is closely related to SWB [4,12,13,14]. For instance, Guardia found secure attachment to be positively correlated with SWB [15]. Ling proved that attachment anxiety and attachment avoidance are both negatively correlated with satisfaction with life and positively correlated with negative affect in college students [16]. Karreman and Vingerhoets found that emotion modulation and mental resilience can mediate the relationship between attachment and SWB among young adults [17].

Athletes are a special group; in China especially, they are involved in sports under a nation-wide system, and the majority of Chinese athletes have lived with their coaches in a relatively closed-off environment from a very young age [18]. The coaches, who are responsible for their athletes’ needs, offer proposals and guidance and support the athletes’ autonomy [19,20]. Sports coaches communicate and encourage their athletes to develop new skills and cope with challenges and provide support platforms for athletes to improve their sports levels. Thus, coaches, as the significant others of athletes, can fulfill the basic attachment functions and inevitably form emotional connections with athletes during extended training and communication, and athletes will regard their coaches as a target of attachment [21]. Studies have shown that mental health and satisfaction with life significantly affect the performance of athletes [22,23]. As has been reported, the attachment of athletes is significantly correlated with the coach–athlete relationship, SWB, attention, and eating disorders [23,24,25]. The coach–athlete attachment scale (CAAS) developed by Davis and Jowett has two versions that respectively consist of 14 items and 19 items. The 14-item version includes two dimensions, namely attachment anxiety and attachment avoidance, and the 19-item version based on the former is supplemented with a 5-item secure attachment subscale [24]. These two versions have both been proven to have high reliability and validity. Later, Davis and Jowett found that coach–athlete attachment can affect the positive and negative affect of athletes via the mediation of the coach–athlete relationship [21]. Milroy et al. found that secure coach–athlete attachment can significantly predict the help-seeking behavior of college athletes [25]. Felton and Jowett discovered that coach–athlete attachment can significantly affect the basic psychological need satisfaction and well-being of athletes [26]. However, there have been few studies on coach–athlete attachment, and the means and underlying psychological mechanism of the effect of coach–athlete attachment on SWB are particularly unclear.

Coach–athlete attachment is a unique type of attachment, so the mechanism of its effect on the SWB of athletes can be explored via the internal working model of attachment [27]. The internal working model theory of attachment holds that attachment, as the emotional link between one and the targets of attachment, affects an individual’s social adaptation and satisfaction with life primarily through the mediation of the internal working model [28], which consists of two components: the perception of social support acquired from others, and the attitude and evaluation of the self [29,30]. The perception of social support acquired from others, namely the perceived social support, refers to the degree of support that is subjectively felt and understood [31]. Previous research has confirmed the correlation between attachment and social support, and individuals with secure attachment report high perceived social support [30,32]. Regarding athletes, research has shown that the perceived social support of athletes is significantly positively associated with athletes’ self-efficacy [33], sports performance [34], strategies for coping with competitive stressors, and SWB [35], and negatively related to psychological responses to injury [36], injury triggers [37], and organizational stressors [38]. Perceived social support further reinforces the safety of athletes, which has been widely analyzed in previous research that describes the manifestations of bullying, harassment, and abuse as a result of the support and concern of coaches regarding athletes and their sports performance [39]. On the other hand, attachment with coaches is extremely important [21,40]. Coaches provide athletes with a growth platform, and encourage them to face new challenges and develop new skills. An insecure coach–athlete attachment may hinder athletes from perceiving their support from coaches. For instance, attachment avoidance can result in a decreased possibility of athletes’ acquisition of coaches’ social support, and attachment anxiety can result in a decrease of the subjective perception of and satisfaction with athletes’ support from coaches [32]. Furthermore, attachment can affect athletes’ attitudes about themselves and self-evaluation; in other words, coach–athlete attachment has a close association with the self-esteem of athletes. Research has shown that the perceived low capability and low value induced by insecure attachment will result in the low self-esteem of athletes [41]. Athletes with secure attachment possess higher self-esteem than those with insecure attachment [42]. It has been proven that attachment anxiety and attachment avoidance can directly affect the self-esteem of athletes [43]. In addition, a study has indicated that social support and self-esteem are also two of the important predictors of SWB [44]. Individuals with sufficient social support have high satisfaction with life, high positive affect, and low negative affect [45]. Naturally, it can reasonably be hypothesized that social support from coaches, as an important component of social support to athletes, can significantly predict the SWB of athletes. Additionally, many studies have proven that those with higher self-esteem are more likely to live in a positive way and thereby have higher SWB [46,47]. The effect of self-esteem on the relationship between perceived social support and well-being has also been investigated [48,49]. For instance, Kong et al. found that self-esteem significantly mediates the effect of perceived social support on well-being in college students [48]. Hence, in the present study, it is also hypothesized that the self-esteem of athletes may mediate the effect of perceived social support on their SWB.

In summation, the current study aims to explore the effects of coach–athlete attachment on the SWB of athletes, and focuses on the mediating roles of athletes’ perceived coach support and self-esteem. The following three hypotheses are proposed:

1. Coach–athlete attachment is significantly correlated with the SWB of athletes;
2. The perceived coach support and self-esteem of athletes are both significantly correlated with their SWB;
3. Both the perceived coach support and self-esteem of athletes can mediate the effect of coach–athlete attachment on their SWB.

## 2. Methods

### 2.1. Participants

A total of 179 athletes from a provincial sports team were enrolled in this study. The participants included divers, gymnasts, table tennis players, weight lifters, and volleyball players. There were 114 males and 65 females. Their ages ranged from 16 to 25 (mean age = 19.12, SD = 2.01), and all participants were national third-class athletes or above. Participants reported an average coach–athlete relationship length of 4.12 years. Altogether, 179 copies of a questionnaire were distributed, and 177 valid copies were returned (2 participants returned the questionnaire without answering all the questions).

The participants voluntarily participated in this survey. This study was approved by the Committee on Human Experimentation of Beijing Sport University (Ethical Code Number: BSU991442).

### 2.2. Instruments

#### 2.2.1. Coach–Athlete Attachment Scale—Fourteen Items (CAAS-14)

This scale was developed by Davis and Jowett, and has been proven to have high reliability and validity [24]. CAAS-14 consists of two subscales, namely attachment anxiety and attachment avoidance, each of which involves 7 items, such as “I worry that I won’t fulfill my coaches’ expectations” and “I do not turn to my coach for reassurance.” The responses for all items were scored on a seven-point Likert scale from 1 (“strongly disagree”) to 7 (“strongly agree”). The mean score of each subscale was expressed as the score of the corresponding dimension, and a higher score indicated a higher level of attachment anxiety or attachment avoidance. The CAAS-14 was translated into Chinese in a previous study, and has exhibited good psychometric properties of validity and reliability [50]. In the present study, the Cronbach’s alpha coefficients for the two sub-scales were 0.81 and 0.72, respectively.

#### 2.2.2. Perceived Coach Support Scale (PCSS)

The sub-scale of the multidimensional perceived social support scale, namely the scale that evaluates perceived social support from significant others, consists of 4 items and was used in the present study [51]. For each item, the term “a special person” was converted to “my coach,” such as, “I can share my joys and sorrows with my coach” and “My coach is a real source of comfort to me.” The responses for all items were scored on a seven-point Likert scale from 1 (“strongly disagree”) to 7 (“strongly agree”). The PCSS was translated into Chinese in a previous study, and has exhibited good psychometric properties of validity and reliability [52]. In the present study, the Cronbach’s alpha coefficient for the PCSS was 0.73.

#### 2.2.3. Rosenberg Self-Esteem Scale (RSES)

This scale compiled by Rosenberg involves 10 items (5 items were scored reversely) with a single dimension. The items include “On the whole I am satisfied with myself” and “All in all, I am inclined to feel that I am a failure.” The responses for all items were scored on a seven-point Likert scale from 1 (“strongly disagree”) to 7 (“strongly agree”). The average score of the items was considered as the score of this scale, and a higher score indicated a higher level of self-esteem [53]. The RSES is commonly used in Chinese, and has been proven to have good reliability and validity [54]. In the present study, the Cronbach’s alpha coefficient for the RSES was 0.86.

#### 2.2.4. Subjective Well-Being Scale (SWBS)

To measure SWB, the three subscales developed by Diener were employed [55]. The life satisfaction subscale comprises five items including “In most ways my life is close to my ideal” and “I am satisfied with my life.” The subscale scores were rated using a seven-point Likert scale ranging from 1 (“strongly disagree”) to 7 (“strongly agree”). The positive affect scale includes six positive emotional words (e.g., “proud”), and the negative affect scale includes eight negative emotional words (e.g., “angry”), both of which were rated using a seven-point Likert scale from 1 (“not at all”) to 7 (“all the time”) in terms of how often the participants experience each emotional state [55]. The SWBS has been widely used in Chinese and has been proven to have good reliability and validity [56]. In the current study, the Cronbach’s alpha coefficients of the three sub-scales of the SWB scale were 0.81, 0.75, and 0.73, respectively. The overall SWB score was determined by adding the standardized scores of the SWLS, positive affect, and negative affect (reversed), with a higher score indicating a higher degree of SWB [57].

### 2.3. Data Analysis

Pearson’s correlation coefficients and hierarchical regression analyses were used to identify relationships among coach–athlete attachment, perceived coach support, self-esteem, and SWB. Baron and Kenny’s recommendations were followed in testing whether perceived coach support and self-esteem mediated the link between coach–athlete attachment and SWB based on hierarchical regression analyses [58]. The following process was conducted. First, a simple regression analysis was performed with the independent variables (attachment anxiety and attachment avoidance) predicting the mediating variables (perceived coach support and self-esteem). Second, a simple regression analysis was conducted with the independent variables (attachment anxiety and attachment avoidance) predicting the dependent variable (SWB). Third, a multiple regression analysis was conducted with the independent and mediating variables predicting the dependent variable. It was observed whether the regression coefficient between attachment and SWB was significantly changed after the introduction of perceived coach support and self-esteem into the model. Then, a structural equation model analysis was conducted to test the mediation effects with Amos 17.0 software. Finally, the bootstrap method was adopted to test the significance of indirect and direct effects in the mediation model [59].

## 3. Results

Table 1 lists the means, descriptive statistics, and intercorrelations of all variables. Attachment avoidance was found to be positively correlated with attachment anxiety (*r* = 0.42, *p* < 0.01) and negative affect (*r* = 0.20, *p* = 0.01), and to be negatively correlated with perceived coach support (*r* = −0.41, *p* < 0.01), self-esteem (*r* = −0.46, *p* < 0.01), life satisfaction (*r* = −0.33, *p* < 0.01), and positive affect (*r* = −0.24, *p* < 0.01). Similarly, attachment anxiety was found to be associated with perceived coach support (*r* = −0.33, *p* < 0.01), self-esteem (*r* = −0.31, *p* < 0.01), life satisfaction (*r* = −0.38, *p* < 0.01), positive affect (*r* = −0.24, *p* < 0.01), and negative affect (*r* = 0.26, *p* = 0.01).

Following the steps of the mediation procedure, a series of simple regression analyses was made, see Table 2. The results indicated that attachment avoidance (β = −0.32, *p* < 0.01) and attachment anxiety (β = −0.20, *p* = 0.01) were each negatively associated with perceived coach support (Model 1), that attachment avoidance (β = −0.40, *p* < 0.01) and attachment anxiety (β = −0.14, *p* = 0.05) were each negatively correlated with self-esteem (Model 2), and that attachment avoidance (β = −0.20, *p* = 0.01) and attachment anxiety (β = −0.28, *p* < 0.01) could significantly predict the overall score of SWB (Model 3). Next, a hierarchical regression analysis was conducted to test the final steps of the mediation procedure. However, when perceived coach support and self-esteem were added to the regression analysis, the relationship between attachment anxiety and SWB (β = −0.19, *p* < 0.01) and that between attachment avoidance and SWB (β = −0.02, *p* = 0.80) became insignificant. According to Baron and Kenny [58], these results indicate partial mediation between attachment anxiety and SWB, and complete mediation between attachment avoidance and SWB.

**Figure 1:**
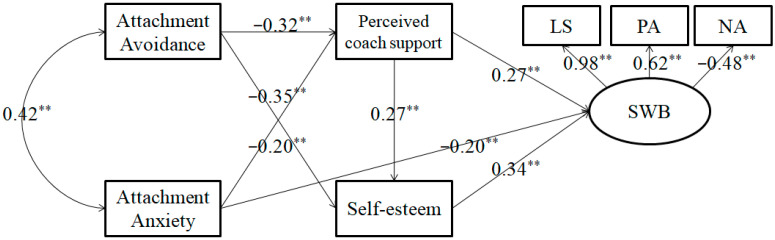
The final model. Note: LS = life satisfaction; PA = positive affect NA = negative affect; ** *p* < 0.01.

Structural modeling analyses were used to further explain the relationships between variables. In the initial model, the independent variables were attachment anxiety and attachment avoidance, the mediating variables were perceived coach support and self-esteem, and the dependent variable was SWB as represented by the observed variables of life satisfaction, positive affect, and negative affect. The initial model hypothesized that both attachment anxiety and attachment avoidance affected perceived coach support, self-esteem, and SWB, that perceived coach support and self-esteem affected SWB, and that perceived coach support affected self-esteem. The initial model was estimated and tested from the structural models using the maximum likelihood method. The results proved that the model fit the sampled data well, but that the paths from attachment avoidance to SWB (*β* = −0.01, *p* = 0.90) and from attachment anxiety to self-esteem (*β* = −0.09, *p* = 0.22) were insignificant. In the final model, the insignificant paths were excluded. The final model was found to fit the sampled data well: χ^2^/df = 2.01, RMSEA = 0.08, SRMR = 0.04, CFI = 0.97 (see Figure 1).

The mediating effects were then tested for significance using the bootstrap estimation procedure in Amos 14.0 software (SPSS Inc., Chicago, IL, USA), a bootstrap sample of 1000 was specified). The 95% confidence intervals of the indirect effect of attachment anxiety on SWB and of attachment avoidance on SWB through perceived coach support and self-esteem were (−0.01 to −0.18) and (−0.13 to −0.35), respectively, and both confidence intervals did not include 0, indicating that the indirect effects were significant. Taken together, these results reveal the mediating roles of perceived coach support and self-esteem in the relationship between coach–athlete attachment and SWB in athletes, thus supporting the hypotheses of this study.

## 4. Discussion

Based on existing attachment theories and targeting athletes, this study was conducted to investigate the effects of coach–athlete attachment on the SWB of athletes, as well as their underlying mechanisms, and some constructive results were acquired. First, it was found that attachment anxiety and attachment avoidance toward coaches were negatively correlated with the SWB of athletes, which is an interesting result that is consistent with the conclusions of other researchers [4,12,13,14]. In the Chinese cultural context, the coach–athlete relationship is an important source of attachment for athletes and has an inevitable effect on the SWB of athletes; as the proverb goes, “one day as a teacher, a life as a father.” Unlike for the typical coach–athlete relationship [28] in which the athlete spends time at their own home and sees the coach for a smaller portion of the day (save for when the team travels), Chinese athletes considered in the current study live together with their coaches all day, and the coach–athlete relationship can also moderately affect the life satisfaction and emotional experience of athletes [21]. Athletes with higher attachment avoidance devote less emotion to their relationships with coaches and maintain spiritual and emotional isolation from coaches. Athletes with higher attachment anxiety, during contact with their coaches, are repeatedly worried about rejection or abandonment by their coaches [10]. This is because they cannot maintain secure and intimate connections with their coaches, feel lower satisfaction with life, and experience more negative affect and less positive affect.

It was also found that coach–athlete attachment was significantly correlated with both perceived coach support and self-esteem. As has been previously reported, high attachment anxiety leads to a contradictory condition during communication and causes anxiety and insecurity, but individuals with high attachment avoidance tend to avoid interpersonal interaction and therefore cannot establish a benign interpersonal relationship network [30,32]. Regarding athletes, whether they are worried about or avoid and escape communication with coaches, they cannot receive enough perceived coach support. Contrarily, athletes with low attachment anxiety and low attachment avoidance can maintain close connections with their coaches and tend to trust them and therefore feel more social support from their coaches [60]. Both attachment anxiety and attachment avoidance were found to be negatively correlated with self-esteem in athletes, which is consistent with the findings of previous studies by other research groups [42,61]. Athletes with secure attachment are more accepting and have more recognition of themselves, evaluate themselves positively, and, in relationships with their coaches, their self-acceptance and thereby self-esteem are higher.

This study focused on the mediating effects of the perceived coach support and self-esteem of athletes on the relationship between coach–athlete attachment and the SWB of athletes. The results of this study support the hypotheses and demonstrate that the effects of attachment anxiety on SWB are partially mediated by perceived coach support and self-esteem, and that the effects of attachment avoidance on SWB are completely mediated by perceived coach support and self-esteem. Moreover, a chain mediating effect was found: coach–athlete attachment → perceived coach support → self-esteem → SWB. Athletes with lower attachment anxiety and attachment avoidance tend to more easily perceive social support from coaches. A high conception of social support promotes individuals to actively establish favorable social relations with others and makes individuals believe that relatives, friends, and significant others will offer them support and help if necessary [62]. Hence, their self-efficacy to face frustration, and thereby their self-esteem, are improved [63]. Regarding athletes, support from coaches helps athletes to form more active self-cognition and thus to buffer the negative life events, pressure, and high-strength training that will impact their SWB. The preceding analyses imply that the two dimensions of the internal operation mode of attachment are not independent in the mechanism of the effect of coach–athlete attachment on SWB. Particularly, perceived coach support will significantly affect athletes’ attitudes about themselves and self-evaluation, namely their self-esteem. The results of the present study suggest that, because the internal working model of attachment is initially formed at childhood, it is very stable and persistent. Hence, it is difficult to improve the SWB of athletes by changing the internal working models of attachment. However, the results suggest that the SWB of athletes can be improved by the increase of their perceived coach support and self-esteem. The findings of the present study highlight the significance of athletes’ self-esteem and social support from their coaches in the relationship between coach–athlete attachment and SWB [21]. Sport psychology consultants can help athletes improve their positive affect and life satisfaction via active self-evaluation and optimistic thoughts. Moreover, the coaches’ care and support for athletes within the coach–athlete relationship is instrumental.

There were some limitations of the present study. First, this cross-sectional study that explored the relationships between coach–athlete attachment and the SWB of athletes cannot clarify the cause–effect relationship between the two. Second, convenient sampling and a relatively small sample size were adopted, and subjects from only one provincial team were recruited; thus, the participants may be homogeneous to some extent, and whether representatives of various sports (i.e., individual, combat, and team sports) and different genders and ages have the same connections with coaches should be speculated with caution. Third, it must be admitted that how an athlete perceives a live-in coach is significantly different from the perception in a typical athletics scenario [38]. This study did not explore the effects of different coaching styles on the perceptions of athletes. It might be interesting in the future to conduct an international experiment and compare the results with those of the present study in terms of the standard error of the mean (SEM). Finally, some potential influencing factors, such as gender and whether the athlete and coach were of the same gender, were not controlled for. It is suggested that these factors be considered in future research to enrich the conclusions.

## 5. Conclusions

1. Significant correlations were found between any two factors including coach–athlete attachment, perceived coach support, self-esteem, and SWB.
2. The insecure coach–athlete attachments, namely attachment anxiety and attachment avoidance, can both significantly negatively predict the SWB of athletes.
3. In line with the internal working model of attachment, the mechanism of how athletes’ attachments affect SWB is as follows: the perceived coach support and self-esteem of athletes both play mediating roles in the effects of coach–athlete attachment on the SWB of athletes.

## Figures and Tables

**Table 1:** Descriptive statistics and correlation analysis of all the variable (*n* = 177).

	Mean	SD	1	2	3	4	5	6
1. Attachment avoidance	3.45	0.8						
2. Attachment anxiety	2.58	0.57						
3. Perceived coach support	4.37	0.62	0.42 **					
4. Self-esteem	3.16	0.33	−0.41 **	−0.33 **				
5. Life satisfaction	4.02	1.06	−0.46 **	−0.31 **	0.41 **			
6. Positive affect	3.9	1.13	−0.33 **	−0.38 **	0.45 **	0.50 **		
7. Negative affect	2.17	0.55	0.20 **	0.26 **	−0.40 **	−0.14	−0.47 **	−0.28 **

Note: ** *p* < 0.01.

**Table 2:** Regression analysis (*n* = 177).

Model	Dependent	Predictors	Model Summary		Coefficients			
			*F*	*R* ^2^	B	SE	*β*	*t*
1	Coach support	Attachment avoidance	21.35 **	0.20	−0.25	0.06	−0.32	−4.28 **
		Attachment anxiety			−0.21	0.08	−0.20	−2.64 *
2	Self-esteem	Attachment avoidance	25.49 **	0.23	−0.17	0.03	−0.40	−5.44 **
		Attachment anxiety			−0.08	0.03	−0.14	−1.89 *
3	SWB	Attachment avoidance	17.77 **	0.17	−0.60	0.23	−0.20	−2.64 *
		Attachment anxiety			−1.19	0.32	−0.28	−3.72 **
4	SWB	Attachment avoidance	19.58 **	0.31	−0.06	0.23	−0.02	−0.25
		Attachment anxiety			−0.81	0.30	−0.19	−2.68 *
		Coach support			1.32	0.28	0.34	4.64 **
		Self-esteem			1.32	0.54	0.18	2.47 *

Note: * *p* < 0.05; ** *p* < 0.01; SWB: Subjective well-being.
